# In the Absence of Animacy: Superordinate Category Structure Affects Subordinate Label Verification

**DOI:** 10.1371/journal.pone.0083282

**Published:** 2013-12-20

**Authors:** Olivera Ilic, Vanja Kovic, Suzy J. Styles

**Affiliations:** 1 Department of Psychology, University of Belgrade, Belgrade, Serbia; 2 Department of Experimental Psychology, University of Oxford, Oxford, United Kingdom; 3 Division of Psychology, Nanyang Technological University, Singapore, Singapore; University of Leicester, United Kingdom

## Abstract

Theoretical accounts as well as behavioral studies reporting animacy effects offer inconsistent and sometimes contradictory results. A possible explanation for these inconsistencies may be inadvertent biases in the stimuli selected for test – with category-specific effects driven by characteristics of test stimuli other than animacy *per se*. In this study, we pit animacy against feature structure (intra-item variability), in a picture-word matching task. For unimpaired adults, regardless of whether objects were from animate (mammals; insects) or inanimate (clothes; musical instruments) superordinate categories, participants were faster to match basic level labels with objects from categories with low intra-item variability (mammals; clothes) than from categories with high intra-item variability (insects; instruments). Thus, pitting animacy against variability allowed us to clarify that observable differences in processing speed between animals and instruments are systematically driven by the intra-item variability of the superordinate categories, and not by animacy itself.

## Introduction

Over the last two decades a series of papers have proposed that animate and inanimate objects are processed differently [1]–[4]. Category-specific processing effects have become a prime source of evidence about the nature of conceptual organization, making the question of what cognitive processes underpin these effects critical for understanding human knowledge structures.

Differences in the processing of animates and inanimates have been taken as evidence for a modular, hierarchical account of semantic organization, in which certain levels of processing are shared by categories which share membership of a higher-order semantic domain or taxonomic structure [1], –[6]. According to these accounts, *cat* and *zebra* would be processed similarly due to shared activation within the semantic domain of animals, while *violin* would be processed differently due to activation in the semantic domain of instruments. Using terminology introduced by Rosch [7], *cat* and *zebra* are basic-level members of the same superordinate category, mammals. Thus if the category of animals happens to be easier to access than the category of fruit, then all members of the category would gain processing advantages via their inclusion in the efficient super-ordinate category.

By contrast, distributed accounts of semantic organization propose that category-specific processing effects may be the outcome of the particular feature-structure of the categories under test, rather than their semantic relatedness. This approach proposes that categories with similar feature-structures (for example, many shared features that frequently co-occur) should be subject to similar processing effects, regardless of whether they share membership of a particular semantic domain [8], [9]. According to this account, *cat* and *zebra* are processed similarly, because both activate a representation within a densely correlated feature-structure (many mammals have two eyes, four legs, fur, etc.), while *violin* is processed differently because its representation has a more sparsely correlated feature-structure (few other instruments have strings, a wooden neck, a carved scroll, etc.), effects which need not rely on higher order semantics. The importance of structural similarity in understanding processing differences between animates and inanimates has also been recognized by other authors addressing this problem from different standpoints [2], [3].

To date, evidence is somewhat mixed as to whether category-specific processing effects are driven by semantic distinctions such as animacy. Evidence supporting a distinction between animates and inanimates originates from neuropsychological studies of patients who exhibit selective impairments in specific object categories like *animals* or *fruits*, alongside unimpaired function in other categories [1], [10]. This dissociation of impairment has been taken as evidence that different superordinate categories are processed in neuroanatomically distinct cortical regions. However, selective deficits have also been observed in patients with less focused neural damage (e.g., Alzheimers: [11], [12]), raising the possibility that selective deficits can arise from unfocussed damage to a more distributed system. Furthermore, while neuroimaging studies have identified regions of activation specific to animates or inanimates [13], [14], these categories also activate common regions, with only small and inconsistent differences between domains [15].

Behavioral studies have also reported category-specific processing effects in unimpaired adults, including differences in speed and accuracy of responses to animates versus inanimates. However, these results are not always straightforward, with some studies reporting an advantage for animates [3], [14], [16], others, an advantage for inanimates [2], [17], [18], and several reporting no differences at all [19]–[21].

One important clue to untangling this pattern of results is that studies comparing only a small number of superordinate categories (e.g., *mammals* vs. *tools*) tend to report animacy-specific processing effects [13], [14], [22], while studies with a more diverse selection of objects report no animacy-specific differences [19], [21], [23]. Thus, it is possible that inconsistencies between reported findings may be due to inadvertent biases in the stimuli selected for test – with category-specific effects driven by characteristics of test stimuli other than animacy *per se*.

One feature which has often been poorly controlled in previous studies is superordinate category feature structure: In particular, animacy has often been confounded with the structure of the superordinate categories under test. When thinking about mammals for example, it is clear that many mammals have similar visual appearance: the vast majority of familiar mammals can be pictured with a horizontal spine, four descending limbs, a head at the front, and a tail at the back – a high density of overlapping visual features. By contrast, the shapes of musical instruments vary greatly, from long, thin flutes to round drums, curvaceous strings, convoluted brass horns, and the block-based shapes of pianos – representing a low density of overlapping visual features. If only mammals (*cat*, *zebra*) and instruments (*violin*) are compared, it is impossible to evaluate whether observed effects arise out of hierarchical semantic organization (animacy), or out of feature-structure alone (high-density versus low-density). In this study we pit semantic taxonomy (animacy) against superordinate category structure (intra-item variability), by selecting four object categories which allow us to independently manipulate the two variables. Furthermore, to clarify the effect of different experimental methods on category-specific processing, we employ three commonly reported behavioural procedures over the same set of test items.

In a picture-word matching task, we expected that structural similarity of categories (e.g. similarity of a cat to a zebra) would influence how rapidly a picture of an object can be correctly matched with its basic level label: We predicted picture-label matching would be influenced by the intra-item variability of the superordinate category, regardless of whether the item is animate or inanimate. In accordance with this view, mammals were expected to be recognized faster than musical instruments, but also faster than insects, because their high structural similarity enhances efficiency in dense rather than sparse distributed networks. Finally, to control for additional sources of stimulus variability, we monitored stimulus typicality and familiarity throughout, along with word frequency for the basic-level labels used in the test.

## Method

### Ethics Statement

This study was conducted in compliance with the guidelines and was approved by the Serbian Psychological Society Research Ethics Committee. All participants gave written informed consent prior to the study.

### Participants

One hundred and five first-year undergraduate psychology students participated in the present study. An additional 20 participants rated the familiarity, and 20, the typicality, of the visual stimuli used in the study. All participants were native speakers of Serbian and received course-credit for participation.

### Stimulus categories and items

The four superordinate categories were selected from the most frequently tested categories from previous studies of animacy effects, for which measures of superordinate category structure were also available: *mammals* (by far most frequent), *birds, body parts, insects, vehicles, clothes, musical instruments, tools, furniture, fruit* and *vegetables*. Many different measures of structural similarity exist, including objective (bottom-up) measures based on physical characteristics of the stimuli [17], [24] and subjective (top-down) measures based on semantic feature production norms [25]. Unfortunately, correlations between these measures tend to be rather small. Even measures based on the same principle can give contradictory results depending on the method of calculation. Although there is no agreement on which of these measures of superordinate category structure are most relevant for understanding processing differences in recognition of objects, one recent paper by Marques and Raposo [26] evaluates 22 bottom-up structural measures of visual stimuli, and their contribution to object decision and object naming latencies. Of four components extracted in their analysis, the one shown to be the best contributor to structural similarity obtained its highest loadings from the bottom-up measure of Euclidean Overlap [17]. The Euclidean Overlap is calculated by measuring the pixel-to-pixel spatial correspondence between pairs of pictures making up the superordinate category. Following the superordinate category variability norms of [17], we selected mammals and insects to represent animates differing in their level of visual similarity, along with clothes and musical instruments to represent inanimates differing in their level of visual similarity. This yielded four superordinate categories for test: Animates with low variability (mammals); animates with high variability (insects); inanimates with low variability (clothes); and inanimates with high variability (musical instruments).

15 nameable basic-level items were selected for each superordinate category under test, alongside 36 filler items (18 animates, 18 inanimates) from a variety of superordinate categories including tools, food, birds, etc., making a total of 96 images. The full list of stimulus items is given in File S1.

In order to further validate the longstanding classification of mammals and certain types of inanimates (here, instruments) as having different levels of item variability (low, high), and to confirm our intuition that the two supplementary categories (insects, clothes) also differed in their level of visual variability (high, low), we subjected the four superordinate categories to an objective assessment of between-item variability, using previously collated picture norms for each of the categories. We collated the Euclidean Overlap of pairs of Snodgrass and Vanderwart line drawings used in Laws and Gales [17] original study, but restricted the analysis to only those items we intended to test.

The level of similarity did not differ significantly between pairs of mammals and pairs of clothes (*χ^2^*(1) = 0.11, *n.s.*), but variability was larger between pairs of insects (in comparison to mammals (*χ^2^*(1) = 10.15, *p*.01) and clothes (*χ^2^*(1) = 8.60, *p*.01)) and musical instruments (in comparison to mammals (*χ^2^*(1) = 13.11, *p*.01) and clothes (*χ^2^*(1) = 12.60, *p*.01)). This confirms that the items selected for test were representative of the broader category norms published by [17].

### Test stimuli

The visual stimuli were full colour photographs of real animate and inanimate objects described above. Photographs of all items were presented on a white background of 280×280 pixels, at a standard screen resolution of 72 ppi. The majority of photographs were from Hemera Photo Objects stock photography, with the remainder from a variety of internet sources. Written words were presented in Serbian using the Latin alphabet, with black text (1 cm high) on a white background. For audio presentation, the 96 labels were digitally recorded and edited to remove background noise, and head and tail clicks using Praat [27].

### Stimulus typicality, familiarity and word frequency

Participants were presented with 60 trials in which a stimulus photograph was accompanied by its written category label (e.g. *zebra*) on a computer screen, and asked to judge on a 7-point scale, either the familiarity of the item, or typicality of the photograph as a member of the labeled category.

Results of the norming study are given in Table 1. Photographs from all four superordinate stimulus categories were judged to be highly typical, with mean ratings above 5.5. Familiarity was more variable across superordinate categories. As both ratings differed between superordinate categories (Familiarity: *χ^2^*(3, *N* = 60) = 464.05, *p*.01; Typicality: *χ^2^*(3, *N* = 60) = 68.32, *p*.01), both typicality and familiarity were included as covariates in the analysis.

**Table 1:** Mean typicality and familiarity ratings.

Animacy	Intra-item Variability	Category	EO (difference)	Typicality *M*(*SD*)	Familiarity *M*(*SD*)
Animate	Low	Mammals	11.72	6.2 (1.3)	4.0 (1.9)
Animate	High	Insects	12.91	5.5 (1.7)	4.8 (1.7)
Inanimate	Low	Clothes	11.57	6.0 (1.7)	6.6 (.9)
Inanimate	High	Instruments	14.26	6.3 (1.2)	3.4 (1.7)

Norms for word frequency of all of the test items in Serbian were collated from the Frequency Dictionary of Contemporary Serbian Language [28].

### Experimental Design

Each image was presented in one of five match types: With its matching label, or one of four mismatching labels. For example, the picture of the zebra could appear with the words *zebra*, ‘zebra’ (match: same animacy, same variability), *magarac*, ‘donkey’ (mismatch: same animacy, same variability), *buba* ‘beetle’ (mismatch: same animacy, different variability), *haljina*, ‘dress’ (mismatch: different animacy, same variability), or *saksofon* ‘saxophone’ (mismatch: different animacy, different variability). The selection of match types was counterbalanced using a Latin Square design. Filler pictures (always labeled correctly) were used to balance the number of match trials. This resulted in a total of 96 trials, with order randomized per participant.

**Figure 1:**
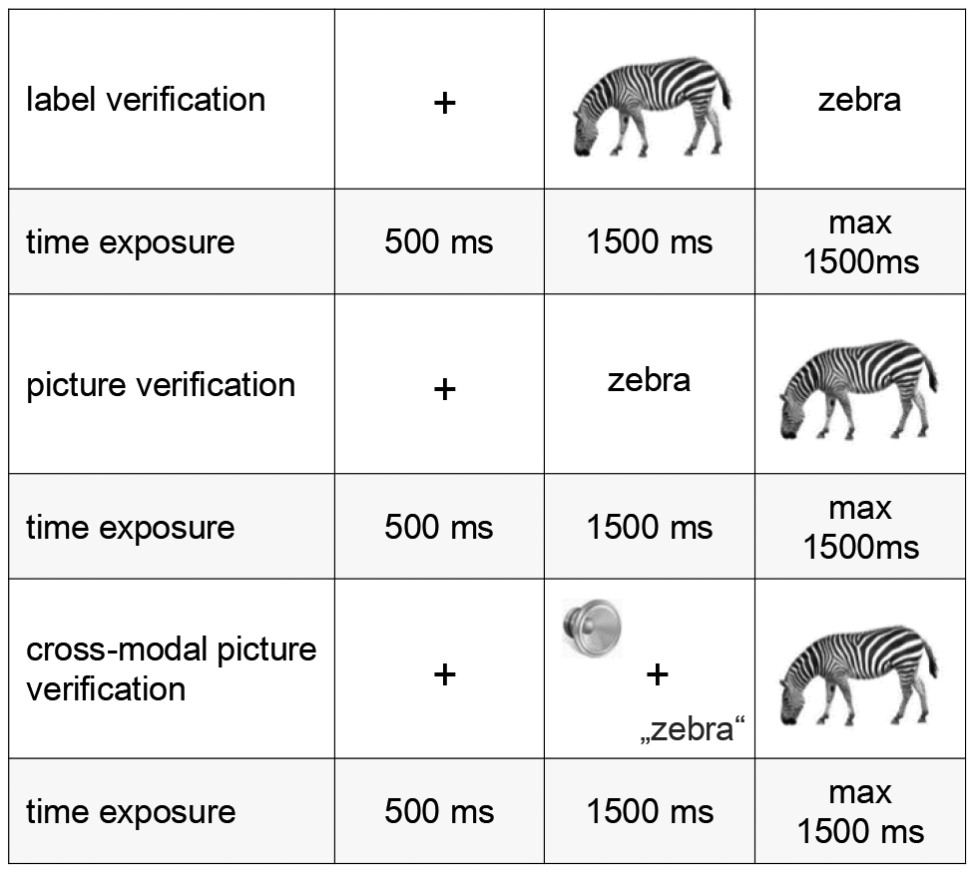
Timing of visual and the auditory stimuli presentation in three procedures.

Three groups of participants (35 participants each) saw the same set of stimuli in one of three procedures: label verification, picture verification or cross-modal picture verification, as illustrated in Figure 1. In all procedures, participants were seated facing a monitor displaying visual stimuli, and in the cross-modal procedure, they listened to audio presented via headphones. Each participant was instructed to judge as quickly and accurately as possible whether the photograph and the word matched. Participants made their responses by pressing one of two mouse buttons, labeled “match” (left) or “mismatch” (right). The selection of mouse buttons was meant to aid participants memory for standard vs. deviant button.

## Results

As overall accuracy was very high (97.5%), error rates were not analysed. For analysis, incorrect trials, trials with no response before the trial end, and trials with extremely short response latencies (200 ms) were excluded, resulting in removal of 3.2% of trials.

In order to approximate normal distribution with minimal a-priori data trimming, RTs were log-transformed [29]. As each participant performed only one of the three tasks, it would be impossible to untangle whether RT differences arise from the differences between the tasks or differences between the participants. To account for this confound of task effects with intra- and inter-individual differences on the distribution of RT, we used standardized log-RTs (For discussion of confound, see [30]; for standardization procedure, see [31]).

As a way for accounting for the ‘language as a fixed effect fallacy’ [32], analysis was conducted using linear mixed-effect modeling [29], [33], [34] with the following entered as fixed effect factors: *animacy* (animate, inanimate), *intra-item variability* (high, low), *task* (label verification, picture verification, cross-modal picture verification), and the composite factor *match condition*, which allowed the five ‘match types’ to be represented by 3 dummy variables: *match type* (match, mismatch), *animacy match type* (mismatch: same, different) and *variability match type* (mismatch: same, different), along with median *typicality*, median *familiarity* of items, and the natural logarithm of *label frequency*. *Participants* and *stimuli* were entered into the model as random-effect factors. As is standard in mixed effect modeling, the *p*-value we report is based on the *t*-distribution, with the number of observations minus the number of fixed-effects coefficients as degrees of freedom. This *p*-value is anti-conservative, especially for small data sets. However, it is the only *p*-value available for the models with random correlation parameters. In the present research, the data set was sufficiently large (*N* = 5753) to minimize anti-conservativeness.

**Figure 2:**
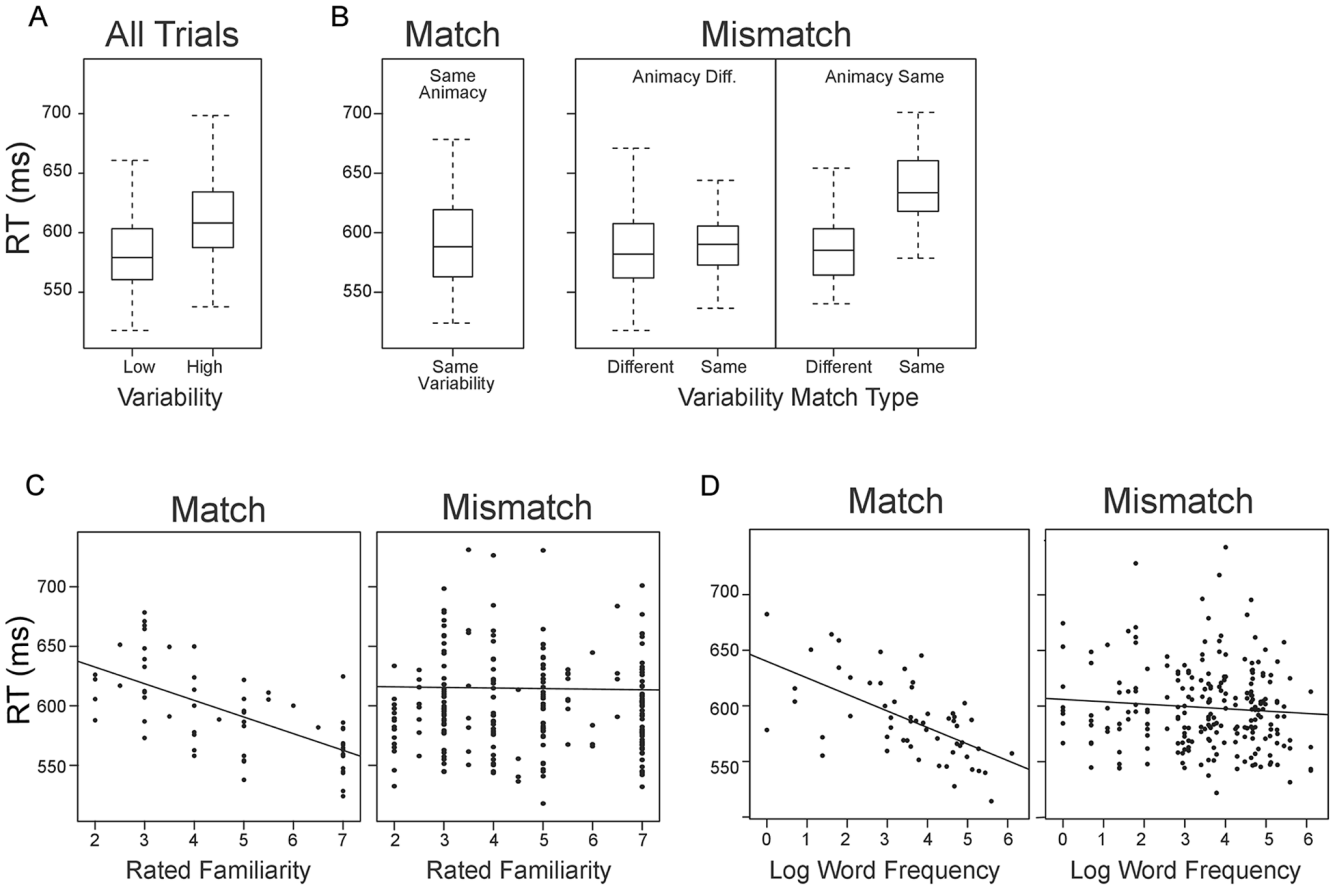
By-item average RTs for significant fixed- and random-effects. A) The graph illustrates significant main effect of intra-item variability. Participants were reliably slower responding to items from the high variability than to items from the low variability superordinate categories. B) The pattern of main effects and interactions concerning the match type variables shows that mismatch from within the same category (e.g., zebra, donkey) caused a substantial RT delay, while other four match conditions RTs were similar. C) and D) The interaction between match-type and word frequency, and match-type and rated familiarity revealed the same pattern of results. Reaction times (RTs) increase with decrease in item familiarity, as well as label frequency, but only in the match condition. There was neither familiarity nor frequency effect for mismatched items. Logarithm label frequency values range from 0 to 6, which corresponds to word frequency from1 to 448. Box plots display median and interquartile range with whiskers depicting upper and lower 5^th^ percentile. Each point in the scatter plots represents one item. Reaction times (RTs) in the graphs are averaged by items and back-transformed to milliseconds.

The full model revealed no significant effect of animacy, neither as a main effect nor in interaction with other fixed- or random-effects. In model fitting procedures of this kind, non-significant predictors are removed in order to find the best fitting model. Thus, animacy was excluded from the final model. The same was true for the task and typicality ratings. After removing potentially influential outliers with absolute standardized residuals exceeding 2.5, the model was refitted. The final model results are summarized in Table 2 and presented in Figure 2.

**Table 2:** Final model results with partial effects for fixed-effect factors.

Effect	Estimate	*t*	*p*
Intercept	−.25	−2.55	.011
variabilityα	.18	4.40	.001
familiarity	.02	1.50	.134
label frequency	−.02	−1.14	.255
match typeβ	.49	5.06	.001
animacy match typeγ	.04	1.20	.229
variability match typeγ	.07	2.30	.021
animacy match typeγ: variability match typeγ	.23	5.29	.001
familiarity: match typeγ	−.05	−3.55	.001
label frequency: match typeγ	−.06	−3.03	.003

α Reference level  =  Low, Contrast  =  High.

β Reference Level  =  Mismatch, Contrast  =  Match.

γ Reference level  =  Different, Contrast  =  Same.

As shown in Table 2, this model identified significant main effects of intra-item variability, match type and variability match type, as well as three significant interactions: between animacy match type and variability match type, match type and familiarity and between match type and label frequency. For the main effect of intra-item variability, the positive value of the estimate (.19) for the difference between the average standardized log-RTs of high and low variability stimuli shows that participants were reliably slower to respond to items from the high variability superordinate categories of insects and instruments than they were to respond to items from the low variability superordinate categories of mammals and clothes. This effect of variability occurs regardless of match type, and was not influenced by any other experimental variables. As is clear in Figure 2 the pattern of effects and interactions concerning the match type variables demonstrated that RTs were similar in the majority of cases, but were substantially slower when people were judging a mismatch drawn from the same superordinate category. The interaction between match-type and rated *familiarity* demonstrated that RT was influenced by item familiarity, generating facilitation for more familiar items when picture and label matched, but not in the mismatch condition. The match-type and word frequency interaction revealed the same pattern, with facilitation for more frequent items, only in the match condition. Finally, there was no significant correlation between word frequency in Serbian and rated familiarity. For the three items (triangle, double bass, xylophone) frequences were less than one per million and were excluded from the primary analisys. Entering dummy values such as 0.001 for these three items gave the same pattern of the results as in the main model, with an inflated interaction between match and label frequency.

In models of this kind, it is not possible to compute standardized effect sizes, however, the overall fit of the model to the data can be assessed using a measure of adjusted R^2^. According to Oberauer Kliegel ([35], page 605, equation 2), the overall fit of the model was robust (*R^2^_Adj._*(5739) = .379).

## Discussion

Regardless of whether items were animate or inanimate, participants were faster to process items from superordinate categories with low intra-item variability (mammals; clothes) than items from superordinate categories with high intra-item variability (insects; musical instruments) – even when other factors such as picture familiarity, typicality, label frequency and match-type were taken into account. Notably, if analysis in the current study had been restricted to just mammals and instruments (common categories for test in the literature on semantic processing), we *might* have been driven to conclude that the observed RT differences were due to the highly salient difference in animacy. However, pitting animacy against variability allowed us to clarify that differences in processing speed between animals and instruments were systematically driven by the intra-item variability of the superordinate categories, and not by animacy itself.

These findings are compatible with theoretical approaches proposing that categories with similar feature-structures (e.g., sharing features that frequently co-occur) are processed similarly, regardless of whether they share membership of a particular semantic domain [8], [9]. According to this view, *cat* and *dress* are processed similarly, because each activates a representation within a densely correlated feature-structure, conferring processing efficiency on both items. By contrast, *violin* and *beetle* are processed differently because their representations reside within more sparsely correlated feature-structures, making them less efficient to access, regardless of higher order semantics. While RT data cannot provide concrete evidence of neural mechanisms in question, differences in RTs are also found in the literature on the neurological disorders where known neurological deficits are accompanied by RT effects [18]. Along these lines, our data provide un alternative explanation for previously reported animacy effects, and suggest that inconsistencies between previous studies exploring animacy effects may be partially explained by peculiarities of the stimuli selected for test: In our study, a categorys intra-item variability was the biggest driver of experimental effects, suggesting that monitoring this variable will be critical in future research into semantic processing.

Even though this study investigates only a small number of superordinate categories, the tightly controlled 2×2 stimulus design allows us to identify whether animacy or variability drive differences in object processing time, either alone, or in combination. In addition, we implemented a statistical approach uniquely suited to extrapolating from only a small set of stimuli: We used linear mixed modeling, which allows both participants and stimuli to be treated as random effect factors. Treating stimuli as a fixed effect factor is especially dangerous when dealing with small sets of stimulus items, since there is a chance that RTs could differ systematically due to extraneous aspects of stimuli. The linear mixed modeling approach helps to avoid this problem. While this approach can be beneficial for any psychological research that attempts to generalize its findings beyond the specific stimulus items, the approach is of particular importance here, because we address the possibility that inconsistencies in previously reported animacy literature may be explained by inadvertent biases in stimuli selected for test.

These results indicate that intra-item variability plays an important role in explaining the recognition speed of living and nonliving objects. In our study this variable was dichotomized into high and low. Although we believe that treating variability as continuous would be preferable, at present there is no single measure of variability which is sensitive enough to capture subtle differences between stimuli which is also a reliable measure of variability. Instead, we implement rough categorization as a proxy. In order to validate the categorization of our stimuli into two groups (high and low similarity), we used the EO norms of pixel overlap between items making up the test set. We selected previously published norms of intra-item EO variability computed by Laws and Gale [17], based on the extensively normed Snodgrass Vanderwart set of line drawings [36]. We based the computation of EO on this picture set for two reasons. Firstly, at present there are no objective measures (mathematical computations) of visual similarity/dissimilarity which are able to quantify similarity between objects depicted by rich, fully-featured photographs, in a way which matches human viewers subjective judgments of object similarity. This is because raw measures of pixel overlap can be heavily biased by aspects of the visual array which are not important for categorization, such as colour variation between members of the same category, or details of local texture. By contrast, when the same computation is applied to line drawings, the measure of EO captures outline information plus some degree of internal structural detail, and this combination appears to best distinguish between categories in comparison with other measures (CO contour overlap, the EO for silhouettes or grayscale versions [17]). We therefore appeal to EO norms of typical exemplars of the items selected for test as a good mathematical approximation of human similarity judgements.

Secondly, although this approach means that the norms for EO variability were computed on a different set of pictures to the photographs used in the test, it should be noted that both line drawings and photographs activate the same meaningful representations of objects depicted (and their labels). It therefore follows that norms based on line drawings still have relevance in defining category level differences between items grouped together. Furthermore, the EO has proven to be in high correlation with performance in different tasks [17], [26], and useful in explaining performance of patients with category specific deficits [17]. We believe that these data demonstrate that validity of the EO measure, as an approximate measure of structural similarity goes beyond explaining processing of the standardized, extensively normed line drawings it was originally calculated on. As we are only using EO to validate a longstanding classification of the relative visual variability of different superordinate categories, we believe that the classification of the four categories into high and low variability is not compromised by this decision.

Interestingly, an alternative measure of conceptual similarity based on semantic feature production norms [25], also groups mammals and clothes together, separate from insects and musical instruments, on the basis of shared features. However, the Cree and McRae norms for category structure were generated in a primarily linguistic task in which participants freely list all semantic features they can think of for a given word. For example, the word *apple* may generate responses such as “can eat it” or “round”. According to this measure, mammals and clothes are higher in variability (.58; .62; these numbers represent the category mean cosine of the angle between the visual feature vectors of each concept pair [25]) than insects and musical instruments (.68; .68) – the opposite to the pattern reported by Laws and Gales [17]. Thus, the use of objective measures (Laws and Gales) versus subjective measures (Cree and McRae) generates contradictory findings in terms of whether a particular category is classed as low or high variability. Despite this apparent contradiction, both studies highlight structural similarities between categories from different semantic domains. The opposite direction of the classifications seems to be a consequence of the different ways in which variability was operationalized, as these measures tap into different kinds of representations at different processing levels. While the Euclidian Overlap is intended to tap into bottom-up visual properties of objects (specifically the similarity of the visual shape of objects), Cree and McRaes measure of similarity is based on top-down knowledge about each items semantic properties as indexed by its linguistic label. Although untangling the dynamics of influence of these two types of similarity in object semantics goes beyond the scope of this study, we believe that it is important to understand that we are possibly dealing with two complementary indexes of semantic processing with different focuses. Our results confirm that variability based on visual norms is a driver of differences in object processing time.

Alongside the key effect of variability, RTs were slower for mismatches where the pairing was from the same superordinate category, indicating a processing cost when rejecting taxonomic sisters. Furthermore, both word frequency in Serbian and the normed familiarity of the test items played a role in RT in the matching condition; matching test stimuli which were frequent or familiar were responded to faster than the average mismatch, while matching stimuli which were low frequency or low familiarity were responded to slower than the average mismatch. Neither frequency nor familiarity had an impact on RT in the mismatching condition.

The fixed allocation of left and right mouse buttons to matching and mismatching trial types somewhat complicates the interpretation of these results. Although included as a memory aid, it is possible that as the left click button (match) is used for most mouse work, this feature of the design may have inflated the difference between the RTs for the match and the mismatch. However, the critical main effect of variability (and lack of main effect of animacy) was evident in spite of this potentially exaggerated RT difference. In fact, the exaggerated difference between match and mismatch RTs may have actually clarified the interactions between match type and rated familiarity and frequency, due to greater sensitivity in the more automated responses using the standard mouse button. Alternatively, it is possible that these interactions arise out of cognitive differences in the verification of matches between labels and objects. Further research using different response paradigms would be needed to clarify which is the driver of these interactions.

One important feature of the current pattern of results is that no interactions were observed between intra-item variability and either item frequency or familiarity – suggesting that the advantage for processing mammals and clothes over insects and instruments was not driven by simple differences between the frequency or familiarity of the test items in those categories, but by structural characteristics of the superordinate category.

In the current study, although three different experimental procedures were used (label verification, picture verification, cross-modal picture verification), no differences between the three tasks were evident. As each task was performed by a different group of participants, standardized RTs were used to remove possible confounds between group differences in performance. However, as there were no interactions between the experimental effects and the task performed, this suggests that the observed effects have equal magnitude in all three experimental tasks (picture-word, word-picture, audio-picture), meaning that they tap into bi-directional linking between pictures and their labels.

Taken together, our results demonstrate that the feature-structure of superordinate categories, along with the familiarity of basic level members and the frequency of their labels are better predictors of processing speed than animacy in unimpaired adults. Furthermore, as stimulus selection can lead to biased interpretations of processing effects, future research into category-specific processing will need to account for the intra-item variability of superordinate categories along with item familiarity and frequency in order to provide clear evidence about the underlying nature of human semantic organization.

## Supporting Information

File S1 (DOC)
