# Correction: In the Absence of Animacy: Superordinate Category Structure Affects Subordinate Label Verification

**DOI:** 10.1371/journal.pone.0100332

**Published:** 2014-06-24

**Authors:** The PLOS ONE Staff

In the Acknowledgments section the authors thanked Professor Petar Milin for his help with statistical analysis and comments in an earlier stage of the project. However, Professor Milin was not consulted in relation to the manuscript submitted to *PLOS ONE* and did not consent to a mention under the Acknowledgments, and therefore, his name should not have been included under the Acknowledgments section. The authors apologize for any inconvenience caused.

The Acknowledgments section should be revised to read:

We would like to thank Professor Keith R. Laws for generously providing us with the Euclidian Overlap data. We are also grateful to Milena Jakic for her assistance during the data collection.

## References

[1] Ilic O, Kovic V, Styles SJ (2013) In the Absence of Animacy: Superordinate Category Structure Affects Subordinate Label Verification. PLoS ONE 8(12): e83282 doi:10.1371/journal.pone.0083282 24376678 10.1371/journal.pone.0083282 PMC3869767

