# Supplementary material for: In the Absence of Animacy: Superordinate Category Structure Affects Subordinate Label Verification
Source: PLoS One. 2013 Dec 20;8(12):e83282. doi: 10.1371/journal.pone.0083282 (PMC3869767; doi:10.1371/journal.pone.0083282)
Supplement: File S1 — (DOC) [file pone.0083282.s001.doc]

Table 1. The full list of stimulus items with their matching and mismatching labels in Serbian

| Object | |  | Label | | | | | |
| --- | --- | --- | --- | --- | --- | --- | --- | --- |
| type | category |  | match |  | mismatch | | | |
|  |  |  | ✓animacy |  | ✓animacy | ✓animacy | ✗animacy | ✗animacy |
|  |  |  | ✓variability |  | ✓variability | ✗variability | ✓variability | ✗variability |
| mammals | bear |  | medved |  | zec | pauk | cipela | daire |
|  | camel |  | kamila |  | lav | osa | jakna | frula |
|  | cat |  | mačka |  | ovca | skakavac | košulja | harmonika |
|  | cow |  | krava |  | medved | vilin konjic | majica | bubanj |
|  | dog |  | pas |  | kamila | komarac | pantalone | flauta |
|  | donkey |  | magarac |  | mačka | moljac | sako | harfa |
|  | elephant |  | slon |  | veverica | leptir | šal | truba |
|  | fox |  | lisica |  | vuk | muva | suknja | metalofon |
|  | horse |  | konj |  | zebra | cvrčak | čarapa | violina |
|  | lion |  | lav |  | slon | svitac | kaput | zvečke |
|  | rabbit |  | zec |  | lisica | bubamara | rukavica | gitara |
|  | sheep |  | ovca |  | konj | bubašvaba | šešir | triangl |
|  | squirrel |  | veverica |  | pas | mrav | čizma | kontrabas |
|  | wolf |  | vuk |  | krava | pčela | džemper | klavir |
|  | zebra |  | zebra |  | magarac | buba | haljina | saksofon |
| insects | ant |  | mrav |  | vilin konjic | lav | daire | cipela |
|  | bee |  | pčela |  | komarac | zec | frula | jakna |
|  | beetle |  | buba |  | moljac | ovca | harmonika | košulja |
|  | butterfly |  | leptir |  | mrav | slon | klavir | čizma |
|  | cockroach |  | bubašvaba |  | muva | mačka | harfa | majica |
|  | cricket |  | cvrčak |  | pčela | lisica | kontrabas | džemper |
|  | dragonfly |  | vilin konjic |  | svitac | magarac | truba | šal |
|  | firefly |  | svitac |  | leptir | medved | bubanj | pantalone |
|  | fly |  | muva |  | buba | konj | saksofon | haljina |
|  | grasshopper |  | skakavac |  | pauk | zebra | triangl | šešir |
|  | ladybug |  | bubamara |  | cvrčak | kamila | flauta | sako |
|  | mosquito |  | komarac |  | bubamara | pas | metalofon | suknja |
|  | moth |  | moljac |  | bubašvaba | krava | violina | čarapa |
|  | spider |  | pauk |  | skakavac | veverica | zvečke | kaput |
|  | wasp |  | osa |  | osa | vuk | gitara | rukavica |
| clothes | blazer |  | sako |  | šešir | violina | ovca | skakavac |
|  | boot |  | čizma |  | majica | truba | krava | leptir |
|  | coat |  | kaput |  | cipela | bubanj | medved | moljac |
|  | dress |  | haljina |  | sako | daire | magarac | komarac |
|  | glove |  | rukavica |  | jakna | flauta | kamila | vilin konjic |
|  | hat |  | šešir |  | košulja | harfa | mačka | muva |
|  | jacket |  | jakna |  | džemper | kontrabas | lisica | pčela |
|  | pants |  | pantalone |  | rukavica | metalofon | zec | osa |
|  | scarf |  | šal |  | čarapa | zvečke | veverica | svitac |
|  | shirt |  | košulja |  | haljina | saksofon | konj | buba |
|  | shoe |  | cipela |  | čizma | klavir | slon | mrav |
|  | skirt |  | suknja |  | suknja | gitara | vuk | bubamara |
|  | socks |  | čarapa |  | šal | triangl | zebra | bubašvaba |
|  | sweater |  | džemper |  | pantalone | harmonika | pas | cvrčak |
|  | t-shirt |  | majica |  | kaput | frula | lav | pauk |
| musical instruments | accordion |  | harmonika |  | harfa | sako | moljac | mačka |
|  | double bass |  | kontrabas |  | gitara | suknja | osa | zec |
|  | drums |  | bubanj |  | klavir | jakna | leptir | krava |
|  | flute |  | flauta |  | kontrabas | košulja | cvrčak | pas |
|  | guitar |  | gitara |  | frula | džemper | pčela | lisica |
|  | harp |  | harfa |  | saksofon | čarapa | muva | slon |
|  | maracas |  | zvečke |  | daire | čizma | mrav | konj |
|  | piano |  | klavir |  | zvečke | šal | pauk | lav |
|  | reed pipe |  | frula |  | flauta | pantalone | komarac | kamila |
|  | saxophone |  | saksofon |  | triangl | cipela | skakavac | ovca |
|  | tambourine |  | daire |  | bubanj | majica | vilin konjic | medved |
|  | triangle |  | triangl |  | harmonika | haljina | buba | magarac |
|  | trumpet |  | truba |  | metalofon | kaput | svitac | veverica |
|  | violin |  | violina |  | truba | šešir | bubašvaba | zebra |
|  | xylophone |  | metalofon |  | violina | rukavica | bubamara | vuk |

Table 2. The full list of filler stimulus items with their matching labels in Serbian

| object | |  | label |  | object | |  | label |
| --- | --- | --- | --- | --- | --- | --- | --- | --- |
| type | category |  | match |  | type | category |  | match |
|  |  |  | ✓animacy |  |  |  |  | ✓animacy |
|  |  |  | ✓variability |  |  |  |  | ✓variability |
| fillers | ball |  | lopta |  | fillers | gorilla |  | gorila |
|  | bed |  | krevet |  |  | hammer |  | čekić |
|  | bench |  | klupa |  |  | hen |  | kokoška |
|  | bicycle |  | bicikl |  |  | kangaroo |  | kengur |
|  | book |  | knjiga |  |  | lamp |  | lampa |
|  | bread |  | hleb |  |  | parrot |  | papagaj |
|  | brush |  | četka |  |  | penguin |  | pingvin |
|  | cake |  | torta |  |  | pipe |  | lula |
|  | cheese |  | sir |  |  | puma |  | puma |
|  | chair |  | stolica |  |  | scissors |  | makaze |
|  | crab |  | rak |  |  | seal |  | foka |
|  | cup |  | šolja |  |  | shark |  | ajkula |
|  | dolphin |  | delfin |  |  | shell |  | školjka |
|  | duck |  | patka |  |  | spoon |  | kašika |
|  | eagle |  | orao |  |  | stork |  | roda |
|  | frog |  | žaba |  |  | tiger |  | tigar |
|  | glasses |  | naočari |  |  | turtle |  | kornjača |
|  | goat |  | jarac |  |  | umbrella |  | kišobran |
